# Complete Genome Sequence of the Endosymbiotic Bacterium “*Candidatus* Riesia pediculicola”

**DOI:** 10.1128/MRA.01181-20

**Published:** 2021-05-06

**Authors:** Jongsun Park, Si Hyeock Lee, Ju Hyeon Kim

**Affiliations:** a InfoBoss, Inc., Gangnam-gu, Seoul, Republic of Korea; b InfoBoss Research Center, Gangnam-gu, Seoul, Republic of Korea; c Research Institute of Agriculture and Life Sciences, Seoul National University, Seoul, Republic of Korea; d Department of Agricultural Biotechnology, Seoul National University, Seoul, Republic of Korea; Indiana University, Bloomington

## Abstract

Human head and body lice host the obligate endosymbiotic bacterium “*Candidatus* Riesia pediculicola.” In this announcement, we describe the complete genome sequence of a “*Ca.* Riesia pediculicola” strain isolated from the human head louse, Pediculus humanus subsp. *capitis*. The inter- and intraspecific variations of endosymbiont genomes were investigated, and this strain was found to display high-level variations in its genome.

## ANNOUNCEMENT

The human head louse, Pediculus humanus subsp. *capitis*, is an obligate hematophagous ectoparasite living on human hair and scalp. It hosts the endosymbiotic bacterium “*Candidatus* Riesia pediculicola,” which is associated with the stomach disc and provides vitamin B, which is lacking in human blood (1).

*P. humanus* subsp. *capitis* specimens were collected in Bristol, United Kingdom, and reared on an *in vitro* rearing system (2). Total DNA was extracted from approximately 500 newly hatched first-instar nymphs before their first blood meal using a DNeasy blood and tissue kit (Qiagen, Hilden, Germany). Genome sequencing was performed on the extracted DNA using the Illumina GAIIx platform at the National Instrumentation Center for Environmental Management (Seoul, South Korea). A total of 114 million 101-bp raw reads (11.5 Gbp) were generated using TruSeq DNA sample preparation kits based on 350-bp DNA fragments. Bioinformatic analyses were performed using default parameters except where otherwise noted. *De novo* assembly of the bacterial genome sequence was performed using Velvet v1.2.10 (3) with the minimum depth option set to 30×, after filtering the raw reads using Trimmomatic v0.33 (4) in the Genome Information System (GeIS) environment. Contigs obtained from the Velvet assembly results were selected if their sequences were matched to those of “*Ca.* Riesia pediculicola” according to BLASTN results against the nonredundant (NR) data set. The gaps were closed using GapCloser v1.12 (5). As the previously sequenced genome (GenBank accession number CP001085) (6) is linear, displaying both end sequences would be repetitive; thus, genome assembly was performed only when the previously assembled genome sequence showed the same phenomenon. After that, all bases were confirmed based on the raw read alignment against the assembled genome using BWA v0.7.17 (7) and SAMtools v1.9 (8). The coverage of this assembled genome sequence is 296.57×. Genome annotation was conducted using the NCBI Prokaryotic Genome Annotation Pipeline (9).

The complete genome sequence of “*Ca.* Riesia pediculicola” is 574,999 bp long, with a GC content of 28.5%. It is slightly larger than that of the previous genome sequence (GenBank accession number CP001085; 574,390 bp). This genome sequence contains 469 protein-coding genes (PCGs), 34 tRNAs, 6 rRNAs, and 1 transfer-messenger RNA (tmRNA). One additional tRNA, *tRNA-Met*, exists in our genome sequence, while the number of PCGs is much smaller because of the putative proteins predicted by GLIMMER.

Based on the pairwise alignment of both genome sequences using MAFTT v7.450 (10), 784 single nucleotide polymorphisms (SNPs) (0.136%) and 80 indel regions covering 669 bp (0.116%) were identified, showing one small inversion in the signal recognition particle receptor FtsY and 16S rRNA (guanine^966^-N^2^)-methyltransferase. The number of SNPs is smaller but the coverage of indels is higher than for Buchnera aphidicola (Aphis gossypii). The proportions of SNPs and indels identified in the two host species genome sequences (1.367% and 0.893%, respectively) (11) are higher than those in the endosymbiotic bacterial genome sequences, suggesting that the pressures of natural selection in the endosymbiont environment may be less than that for the host species. This is similar to the case between the mitogenomes of Aphis gossypii (12, 13) and its endosymbiont.

Our “*Ca.* Riesia pediculicola” genome sequence can provide insight into the intraspecific variation of endosymbiont genomes, as well as clues to understanding their evolutionary histories, with additional endosymbiont genome sequences forthcoming.

### Data availability.

The “*Ca.* Riesia pediculicola” whole-genome sequencing project has been deposited at DDBJ/ENA/GenBank under accession number CP062474, BioProject accession number PRJNA665755, and BioSample accession number SAMN16268218. The raw sequences were deposited under accession number SRR12717033.
